# A Compend of Electricity, and Its Medical and Surgical Uses

**Published:** 1887-07

**Authors:** 


**A Compend of Electricity, and its Medical and Surgical Uses.** By CHAS. F. MASON, M.D., Assistant Surgeon U. S. Army, and CHARLES H. MAY, M.D., N. Y. Polyclinic. Philadelphia: P. Blakiston Son & Co., 1012 Walnut street. 1887.

This useful little work is No. 3 of the "Medical Brief" series. It presents a short yet clear view of an important and generally ill-understood branch of therapeutics. A mastery of the facts and principles here taught will serve as an admirable introduction to the study of the larger works on this subject.

